# Prevalence and determinants of antepartum depressive and anxiety symptoms in expectant mothers and fathers: results from a perinatal psychiatric morbidity cohort study in the east and west coasts of Malaysia

**DOI:** 10.1186/s12888-018-1781-0

**Published:** 2018-06-15

**Authors:** Hashima E. Nasreen, Jamalludin Ab Rahman, Razman Mohd Rus, Mira Kartiwi, Rosnah Sutan, Maigun Edhborg

**Affiliations:** 1 Faculty of Medicine, International Islamic University Malaysia, Jalan Sultan Ahmad Shah, 25200 Kuantan, Pahang Malaysia; 2 Faculty of Information and Communication Technology, International Islamic University Malaysia, Jalan Gombak, 53100 Kuala Lumpur, Malaysia; 3 Community Health Department, Universiti Kebangsaan Malaysia, Bandar Tun Razak Cheras, 56000 Kuala Lumpur, Malaysia; 4 Department of Neurobiology, Care Sciences and Society, Karolinska Institute, SE-141 83 Huddinge, Stockholm, Sweden

**Keywords:** Antepartum, Depressive symptoms, Anxiety symptoms, Associated factors, Expectant mothers and fathers, Malaysia

## Abstract

**Background:**

Research on antepartum psychiatric morbidities investigating depressive and anxiety symptoms in expectant mothers and fathers is lacking in low- and middle-income countries. This study aimed to estimate the prevalence of antepartum depressive, anxiety and co-occurring significant symptoms and explore the associated factors in a cross-section of Malaysian expectant mothers and fathers.

**Methods:**

We used cross-sectional data from a prospective cohort study of 911 expectant mothers and 587 expectant fathers during their third trimester of pregnancy, from health clinics of two states in the east and west coasts of Malaysia. The validated Malay version of Edinburgh Postnatal Depression Scale and the anxiety sub-scale of Depression, Anxiety and Stress Scale were used to measure the depressive and anxiety symptoms. Multiple logistic regression analyses identified the determinants of antepartum depressive and anxiety symptoms (ADS and AAS).

**Results:**

Prevalence of ADS was 12.2% in expectant mothers and 8.4% in expectant fathers, while AAS was 28.8% in expectant mothers and 13.3% in expectant fathers, and co-occurring significant symptoms was 8.0% in expectant mothers and 4.0% in expectant fathers. Expectant mothers and fathers having perceived social/family support were less likely to suffer from ADS. Intimate partner violence, poor relationship with husbands, depression in earlier pregnancy and husband’s depression in current pregnancy in expectant mothers, and living in rented house, sex preference for the unborn child, stressful life events and wife’s depression in current pregnancy in expectant fathers were associated with a greater likelihood of ADS. The determinants for AAS were living in rented house and with parents/in-laws, poor relationship with husbands, restrictions during pregnancy and stressful life events for expectant mothers, and stressful life events and being unsupportive towards wives in household chores for expectant fathers.

**Conclusion:**

Both ADS and AAS are prevalent in expectant mothers and fathers, and largely an undetected problem in Malaysia. Administration of couple-based screening and referral program during antenatal check-up should be universal practices to identify and treat the psychiatric morbidities.

## Background

High maternal perinatal depressive and anxiety symptoms are traditionally evident [1]. It is widely believed that only mothers are affected by depression during pregnancy and postpartum period. Therefore, most research are focused on mothers. However, contemporary research findings, mostly from high-income countries, suggest that fathers are also affected by depression during ante and postpartum period [2]. Recent research findings in high-income countries show that anxiety is more prevalent than depression among mothers during their perinatal period [1, 3]. Moreover, anxiety and depressive symptoms are more prevalent during pregnancy than postpartum period both in mothers [1, 4] and fathers [1, 5]. Meta-analysis indicates the mean weighted prevalence for maternal antepartum depressive symptoms (ADS) is 12% in high income countries [6] and about 20% in Asia [7]. The meta-estimation of paternal ADS was 8% with significant heterogeneity observed among prevalence rates ranging from 2 to 19% [2, 4]. Emerging literature reported that 18–25% of expectant mothers [1, 3] and 8–20% of expectant fathers showed antepartum anxiety symptoms (AAS) [1]. Comorbid depressive and anxiety symptoms in mothers are common during pregnancy [1, 8], however, very few data are available about expectant fathers [1].

The prevalence of ADS and AAS tends to be higher in low- and middle-income countries (LMIC) than in high-income countries [9, 10]. Although the World Health Organization (WHO) ranks depression as the leading cause of disease burden for women of childbearing age (15–44 years) [11], often both ADS and AAS go unrecognized and untreated in LMIC [12]. However, ADS has impact on infants, such as low birth weight [13], premature delivery [14], poor nutrition [15], difficult temperament, impaired cognitive development and conduct disorder during childhood [16], and more direct impact on mothers, such as impaired mother-infant relationship [17], poor partner relationship [12], alcohol and substance use [18], the postpartum depression itself and suicidal ideation [19]. Evidences from high-income countries indicate that maternal AAS has similar effects on children’s emotional, behavioral and physical development as do maternal depression [20]. Paternal ADS is negatively associated with intimate relationship satisfaction [2], parenting practices [21], and emotional and behavioral outcome of children [22].

Risks for maternal ADS differ between high-income countries and LMIC [8, 12]. Studies from Asian countries reported that risk factors for maternal ADS included socioeconomic disadvantage, unintended pregnancy, intimate partner violence (IPV), lack of social and family support, life stress, insufficient emotional and practical support, having hostile in-laws, sex preference for the unborn child, and history of mental illness [12, 23, 24]. A systematic review of 97 papers indicated that the risk factors for maternal AAS are analogous to the risk factors of maternal ADS [25]. However, the risk factors for paternal ADS are poorly understood. Based on the systematic review by Wee et al. [2], the most common correlate for paternal ADS were having a partner with elevated depressive symptoms and poor relationship satisfaction. Similar evidence is lacking in LMIC.

Research in Asia including Malaysia mostly reported on maternal postpartum depression, a few on maternal ADS [10, 26] and AAS [10, 23]. Data on expectant fathers are not available. ADS and AAS have not yet been investigated together and simultaneously for expectant mothers and fathers in Malaysia. This study aimed to estimate the prevalence and to identify the determinants of ADS and AAS in expectant mothers and fathers. The study also compared the prevalence of ADS, AAS and co-occurring significant symptoms between expectant mothers and fathers in the east and west coasts of Malaysia.

## Methods

### Study design and setting

Data for this cross-sectional study were originated from the baseline phase (third trimester of pregnancy) of a larger prospective cohort study of parents and their infants, which aimed to assess the impact of parental perinatal depressive and anxiety symptoms on infants’ growth and development. The prospective cohort study recruited expectant mothers and fathers during the third trimester of pregnancy, and followed up mothers, fathers and infants at birth, 2–3 and 6–8 months postpartum on parents’ depressive and anxiety symptoms and infants’ growth and development. The study was conducted in health clinics in Pahang and Selangor states in the east and west coasts of the peninsular Malaysia, respectively. The population in Pahang is largely rural, religious and poor, where the economic mainstay is agriculture and mining contributing 4.2% to country’s gross domestic product (GDP). The majority of women in Pahang are involved in unpaid domestic work including child care. On the contrary, Selangor is one of the most developed and populous state which is predominantly urban and contemporary. The major sources economy in Selangor are manufacturing and services sectors that contributed 22.6% to GDP (27). Pregnant women in Malaysia get free antenatal and postnatal care from government health clinics and hospitals, and majority of the births occur at hospitals. Whatever they plan to give birth in a government or private hospital, they can register and attend periodic check-ups at the nearest government health clinics.

### Sample

Considering a prevalence of maternal depression of 21–22% [26, 27], ratio between exposed and unexposed is 1:5, prevalence of child outcomes on underweight and wasting 12% and stunting 16% [28], a significance level of 5% and a power of 80%, the estimated sample size for the larger prospective cohort study was 586 women (98 from exposed and 488 from unexposed). Taking into account a non-response rate of 30% and attrition rate 20%, the calculated sample size at baseline was 900 expectant mothers and fathers. To attain the required sample, 10 health clinics (four from west coast and six from east coast) were selected, where the highest number of attendance for antenatal check-up was observed.

Expectant mothers and fathers who attended the health clinics for their routine antenatal care at the third trimester of pregnancy, in other words when the length of pregnancy is 7 months and onward, were invited to participate in this study. Exclusion criteria for the original cohort study included non-Malaysian, illiterate, multiple pregnancy (as it may affect the child outcome) and intrauterine death. The trained clinic nurses explained the aims and procedures of the study, and respondent’s right to refuse to participate or to terminate the interview at any point. Of the 911 couples approached, 904 expectant mothers (454 from east coast and 450 from west coast) and 587 expectant fathers (440 from east coast and 147 from west coast) signed the informed consent and agreed to take part indicating participation rate of 99 and 64% in expectant mothers and fathers, respectively.

### Data collection

Data for the prospective cohort study were collected during March 2016 to August 2017. Data at baseline (third trimester of pregnancy) include information on socioeconomic condition, reproductive health, perceived social support, intimate partner violence (IPV), stressful life event, previous/family history of depression, and depressive and anxiety symptoms. Data from expectant mothers were collected through self-reported structured questionnaire at the health clinics. Information from expectant fathers was collected in the same manner in a separate room, if they accompanied their wives in the clinics. Questionnaires and informed consent forms were given to expectant mothers whose husbands did not accompany them to clinics. The expectant fathers who agreed to participate signed the informed consent and filled out questionnaires, and sent them back to the respected health clinics by their wives during subsequent visits. The clinic nurses and research assistants received training on the questionnaire and data collection procedures, and scrutinized the filled in questionnaires for any missing data. The questionnaire was pretested with pregnant women in a clinic other than the study sites, and was revised based on feedback received in the field test.

### Assessment of explanatory variables

Age of the respondents was calculated in years. Socioeconomic status was indicated by respondent’s educational level (primary, secondary or tertiary), income earner (yes or no), occupation (government employee, non-government employee, self-employed or unemployed/homemaker), monthly household income and living condition (own house, rental house or living with parents/in-laws). Based on the monthly household income, the household was ranked as high (>RM 5599), middle (RM 2300–RM 5599) or low income (<RM 2300) [29]. The obstetric indicators encompassed parity (primi or multipara), number of children, whether the current pregnancy was planned or unplanned, and preferences for gender on unborn child. Facing restriction during pregnancy was assessed if the expectant mothers were confronting any involuntary dietary restriction or movement restriction or both during pregnancy.

IPV was indicated by a lifetime experience of physical violence ever by the husband, forced sex ever, and physical violence during pregnancy. Physical violence included being slapped, shoved, punched, kicked or dragged on the ground by the partner [30]. The total score of physical violence was ranging from 0 to 4, and categorized as no act of physical violence (0) and acts of physical violence (1–4). Respondent’s perceived relationship with spouse was measured as categorical and dichotomized as good (included very good and good) and poor (included not so good and bad). Expectant fathers’ support towards their wives in household chores was considered as a proxy indicator of good spousal relationship.

Perceived social support was assessed by the validated Malay version of Multidimensional Scale of Perceived Social Support (MSPSS) [31]. MSPSS included 12 items, scored on a 7-point scale from 1 (very strongly disagree) to 7 (very strongly agree). The scale was categorized as low support (mean score 1–2.9), moderate support (mean score 3–5) and high support (mean score 5.1–7). MSPSS comprised of three subscales, including family support (4 items), friends’ support (4 items) and significant other support (4 items), a higher score indicating more support [32]. The scale demonstrated good internal consistency in the present study with the Cronbach’s alpha between 0.87 and 0.94 on the three subscales.

Stressful life event was assessed by six questions, scored 1 (yes) or 0 (no): 1) any life-threatening illness, 2) any life-threatening accident, 3) loss of family members/friends that affects daily living activities, 4) any event/torture/abuse that emotionally putting down, 5) severe financial difficulty, 6) any other situation that extremely frightening or horrifying in which they felt extremely helpless. A scale was composed ranging from 0 to 6, and dichotomized as 0 indicating experiencing no stressful life events and 1–6 experiencing stressful life events.

### Measurement of depressive symptoms

The Edinburgh Postnatal Depression Scale (EPDS) was used to detect depressive symptoms among parents during pregnancy [33]. The EPDS is a 10-item self-administered questionnaire, where each item is rated on a 4-point scale (0–3), with total score ranging from 0 to 30. The scale rates the intensity of depressive symptoms in the last 7 days where higher score indicates more depressive symptoms. The items assessed dysphoric mood (five items), anxiety (two items), guilt (one item), ability to cope with everyday life (one item), and suicidal thought (one item). The reliability and validity of the Malay version of the EPDS was verified, whereby a score of 11.5 represented the optimum cut-off point for 72.7% sensitivity, 95% specificity and 80% positive predictive value [34]. Therefore, expectant mothers with an EPDS score ≥ 12 was categorized as having depressive symptoms and ≥ 16 as severe depressive symptoms in this study. The EPDS was also validated for use among expectant fathers, and the recommended cut-off point is ≥10 for them [35]. The scale presented good reliability in the study with Cronbach’s alpha of 0.75.

### Measurement of anxiety symptoms

General anxiety was assessed using the validated Malay version of the anxiety sub-scale of Depression, Anxiety and Stress Scale (DASS 21) [36]. It was used to screen and measure the level of anxiety over the previous week. DASS 21 comprised of 21 questions, which are subdivided into three domains with seven questions in each, to represent domains of depression, anxiety and stress. The choice of response ranged from 0 (‘did not apply at all’) to 3 (‘applied very much’ or ‘most of the time’). In this research we used only the anxiety scale items of 2, 4, 7, 9, 15, 19 and 20. As we used the short version of DASS (21 v 42 items), score on the DASS 21 anxiety was multiplied by 2 to calculate final score of anxiety symptoms [37]. The cut-off point used in the study was ≥8 to estimate the prevalence and ≥ 15 to estimate severe anxiety symptoms [37]. The DASS 21 anxiety scale showed a good reliability in this study with Cronbach’s alpha of 0.74.

Co-occurring significant symptoms were assessed if the expectant mothers scored ≥12 on EPDS and ≥ 8 on DASS 21 anxiety scale, and expectant fathers scored ≥10 on EPDS and ≥ 8 on DASS 21 anxiety scale.

### Analysis

Descriptive analyses were performed for background characteristics of the respondents and prevalence of ADS and AAS. Point prevalence of ADS and AAS during third trimester of pregnancy was calculated by dividing the number of cases by the total number of non-missing outcome data at that time. An independent t-test was used to compare means of age, family support, friends’ support and significant other support of MSPSS between expectant mothers and fathers. Bivariate analyses (*χ*^*2*^ test, Fisher’s exact test and Pearson’s point bi-serial correlation coefficient) were conducted between each independent variable and the outcome variables ADS and AAS independently to identify the possible contributory factors. The independent variables with *p* < 0.05 were considered as possible contributory factors and included in the multiple logistic regression enter models. The outcome variables i.e. ADS and AAS were measured as both numerical and dichotomous. However, as there were well-known and recommended cut-off points on both variables, we used the outcome variables as dichotomous in multiple logistic regression. Subsequently a final model was emerged using only the significant variables ascertained in multiple regression models. We reported an odds ratio (OR) at 95% confidence level to indicate the likelihood of reporting depressive and anxiety symptoms. Any violation of assumption was observed by examining the interaction between explanatory variables in the models.

## Results

### Sample profile

The final sample included 904 expectant mothers (50.2% from west coast and 49.8% from east coast) with a mean age of 29 years and 583 expectant fathers (24.5% from west coast and 75.5% from east coast) with a mean age of 32 years in the third trimester of pregnancy after excluding four men because of missing outcome data. Expectant mothers were more educated than fathers in east coast, while no difference was observed in west coast. Half of the expectant mothers in east coast and one-third in west coast were homemakers. More expectant fathers were employed in private or public sector in west coast than in east coast. Nearly all the participants (> 90%) were Muslim and the majority were Malay (Table 1). Approximately 53% of the participants were from middle income level with median monthly household income of RM 3300 and 26% from low income level with significantly higher proportion fitted in east coast (36.5%) than in west coast (16.2%). Three quarters of the respondents were living in nuclear family with husband and children, amongst 60% lived in rented houses (data not shown).

**Table 1:** Background profile of the respondents at third trimester of pregnancy (in percent unless otherwise specified)

	East Coast			West coast				
	Mothers *N*^a^=450	Fathers *N*^a^=440	*p* value	N^a^	Mothers *N* = 454	N^a^	Fathers *N* = 143	*p* value
Socioeconomic profile								
Age, mean (SD)	29.3(5.0)	32.3(6.1)	< 0.001	448	28.9(4.1)	134	30.1(5.1)	0.005
Race				453		138		
Malay	94.7	82.7	< 0.001		91.8		89.9	0.469
Chinese/Indian/aboriginal	5.3	17.3			8.2		10.1	
Religion				453		138		
Islam	94.9	94.8	0.943		92.5		94.9	0.325
Hindu/Buddhist/Christian	5.1	5.2			7.5		5.1	
Education				449		132		
Primary	4.0	4.1	0.012		0.4		2.3	0.136
Secondary	52.0	61.6			31.6		31.1	
Tertiary	44.0	34.3			67.9		66.7	
Occupation				454		134		
Government employee	24.0	26.1	< 0.001		19.6		20.9	< 0.001
Non-government employee	19.8	41.8			37.0		57.5	
Self employed	5.6	31.8			9.0		21.6	
Home maker/unemployed	50.7	0.2			34.4		0.0	
Obstetric information								
Parity				454				
Primipara	31.8	–			40.1		–	
Multipara	68.2				59.9			
Planned current pregnancy	49.1	53.9	0.156	449	45.0	129	45.0	0.996
Sex preference				449		131		
Boy	14.4	27.0	< 0.001		19.4		28.2	< 0.001
Girl	16.7	12.3			17.8		4.6	
No preference	68.9	60.7			62.8		67.2	
Social support				453		143		
Low	1.3	0.9	0.083		0.9		0.0	0.090
Moderate	35.1	42.3			23.4		31.5	
High	63.6	56.8			75.7		68.5	
Family support, mean (SD)	22.5(3.5)	21.8(3.7)	0.004		23.4(3.5)		22.4(3.9)	0.003
Friends support, mean (SD)	20.2(4.1)	19.7(3.9)	0.079		20.9(3.9)		19.5(4.1)	< 0.001
Significant other support, mean (SD)	22.9(4.5)	22.6(4.5)	0.412		23.6(4.2)		24.2(4.5)	0.151
Intimate partner violence								
Poor relations with spouse	15.6	14.1	0.213	452	22.1	132	4.5	0.012
Physical abuse ever	2.7	–		452	2.7		–	
Physical abuse during current pregnancy	1.3	–		453	1.5		–	
Experienced stressful life event	13.6	20.2	0.008	452	18.1	142	35.2	< 0.001
Previous history of mental illness				448		126		
Previous depressive symptoms	3.6	2.0	0.173		12.7		7.1	0.083
Depressive symptoms in earlier pregnancy	6.7	2.7	0.006		12.3		8.0	0.182

^a^Data available for analysis

No significant differences were observed between the expectant mothers whose spouse agreed or disagreed to participate in the study in terms of age, race, religion and parity. However, a higher percentage of expectant mothers whose spouse did not agree to participate were from west cost (*χ*^*2*^ = 555.53; *p* < 0.001), richer (*χ*^*2*^ = 39.789; *p* < 0.001), more likely to be income earner (*χ*^*2*^ = 13.993; *p < 0*.001) or empolyed (*χ*^*2*^ = 28.891; *p* < 0.001), and had a tertiary level of education (*χ*^*2*^ = 26.067; *p* = 0.001).

### Obstetric profile

Almost 36% of the respondents were primiparas with higher proportion fitted in west coast (*χ*^*2*^ = 6.778; *p =* 0.009). The mean number of children was two. Six percent of the parents experienced the death of 1–2 children, including stillbirth; and 18% of the mothers experienced abortion. Three out of every 10 expectant mothers faced cultural restrictions related to food and/or movement during pregnancy with higher proportion (*p* = 0.001) in west coast (36%) than in east coast (25.8%) (data not shown). Half of the respondents stated that the current pregnanacy was planned. More expectant fathers than expectant mothers expressed a desire to have son in both settings (Table 1).

### Social support, intimate partner violence and stressful life event

Expectant mothers received higher perceived social support than expectant fathers, with a mean score of 23.0 (SD 3.5) for expectant mothers and 21.9 (SD 3.7) for expectant fathers on MSPSS-family support, and 20.5 (SD 4.0) for expectant mothers and 19.6 (SD 3.9) for expectant fathers on MSPSS-friend’s support (data not shown). However, no significant difference between expectant mothers and fathers was noted in perceived social support after stratification by sites. More than 90% of the expectant fathers support their wives in household chores. Three percent of the expectant mothers in both west and east coasts reported of being the victim of at least a single act of IPV ever, including 1.4% (*n* = 13) reporting physical abuse during the current pregnancy (Table 1). About 2 % of the expectant mothers experienced of being forced to have sex with their spouse (data not shown). One-fifth of the expectant mothers and one-tenth of the expectant fathers reported that their relationship with their spouses was poor, with significantly higher proportion fitted in west coast. Conversely, more expectant fathers than expectant mothers experienced single or multiple stressful life events in their life. Overall, expectant mothers were more likely to report depression in earlier pregnancy and history of previous depressive symptoms than expectant fathers (Table 1).

### Prevalence of antepartum depressive and anxiety symptoms

**Fig. 1:**
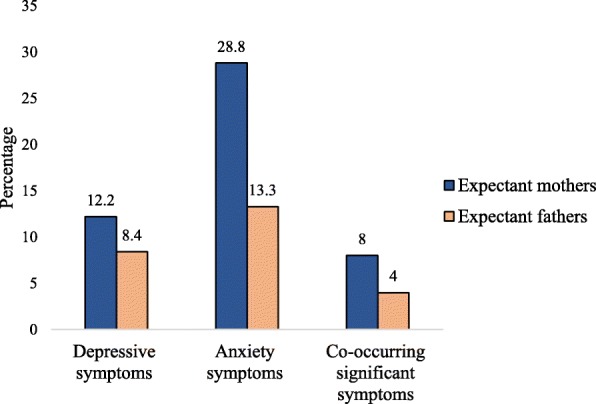
Prevalence of parental antepartum depressive and anxiety symptoms

Of the 904 expectant mothers assessed during their third trimester of pregnancy, 110 had EPDS scores ≥12 and 206 had DASS 21 anxiety scores ≥8 indicating an ADS point prevalence of 12.2% (CI_95_ 10.7–13.7%) and AAS point prevalence of 28.8% (CI_95_ 25.8–31.8%). Similarly, the point prevalence of ADS was 8.4% (CI_95_ 6.1–10.7%) and AAS was 13.3% (CI_95_ 10.5–16.1%) in expectant fathers. The expectant mothers had higher rate of ADS (*χ*^*2*^ = 5.26, *p* = .022) and AAS (*χ*^*2*^ = 48.64, *p < 0*.001) than expectant fathers. Similarly the expectant mothers had twice as high the prevalence of co-occuring significant symptoms than expectant fathers (*χ*^*2*^ = 49.06, *p*.0 < 001) (Fig. 1). The point prevalence of AAS was found to be higher in west coast than in east coast in both expectant mothers (34.0% vs. 23.6%, *p* = .001) and fathers (18.4% vs. 11.6%, *p* = .037). However, no differences were noted between two sites in the prevalence of ADS (13.0% in west cost vs. 11.0% in east coast, *p* = .445 in expectant mothers, and 9.8% in west coast vs. 8.0% in east coast, *p* = .492 in expectant fathers) and co-occurring significant symptoms (8.2% in west cost vs. 7.8% in east coast, *p* = .829 in expectant mothers, and 3.5% in west coast vs. 4.1% in east coast, *p* = .773 in expectant fathers) in expectant mothers and fathers (data not shown).

Twenty-eight expectant mothers (3.1%) and seven expectant fathers (1.2%) (*p* = .019) scored ≥16 on EPDS, and 39 (4.3%) expectant mothers and nine expectant fathers (1.5%) (*p* = .009) scored ≥15 on DASS anxiety. Seven depressed expectant mothers and one depressed expectant father thought of harmimg themselves quite often (data not shown).

### Determinants of antepartum depressive and anxiety symptoms

After adjusting with possible associated factors, the multiple logistic regression models showed both protective and risk factors for ADS among the expectant mothers and fathers. The protective factors included high perceived social support for expectant mothers and higher family support for expectant fathers (Tables 2 and 3). Maternal depressive symptoms were independently and positively associated with IPV ever, poor relationship with husband, depression in earlier pregnancy, and husband’s depression in current pregnancy (Table 2). On the contrary, the risk of paternal depression increased with living condition (if living in a rented house), son preference, stressful life event, and depression during wife’s earlier pregnancy (Table 3). No significant interaction between explanatory variables was found. The Hosmer and Lemeshow tests (*p* = .281 for expectant mothers’ model and *p* = .748 for expectant fathers’ model) indicated that the models fit the data well. Site was not included in multiple logistic regression as it was not associated with ADS in bi-variate analysis in both expactant mothers and fathers.

**Table 2:** Logistic regression model examining associated factors of expectant mothers’ depressive symptoms

Associated factors	Full model		Final model	
	Adjusted OR	CI_95%_	Adjusted OR	CI_95%_
Socioeconomic status				
Income earner				
No	1			
Yes	1.08	0.51–2.26	–	
Economic status				
High income	1			
Middle income	1.07	0.44–2.59	–	
Low income	1.55	0.53–4.49	–	
Social support				
Low support	1		1	
Moderate support	0.12	0.02–0.65	0.16	0.03–0.73
High support	0.09	0.02–0.50	0.13	0.03–0.59
Intimate partner violence				
Experienced physical violence ever				
No	1		1	
Yes	10.93	2.79–42.83	12.13	3.48–42.23
Relationship with husband				
Good	1		1	
Poor	3.50	1.78–6.87	3.92	2.05–7.53
Pregnancy related indicators				
Planned current pregnancy				
No	1			
Yes	0.65	0.35–1.21	–	
Husband’s sex preference				
No	1			
Yes	1.18	0.62–2.23	–	
Restriction during pregnancy				
No	1			
Yes	1.73	0.90–3.35	–	
Stressful life event				
No	1			
Yes	1.86	0.89–3.88	–	
History of mental illness				
Previous history of depression				
No	1			
Yes	0.96	0.31–2.98	–	
Depression in earlier pregnancy				
No	1		1	
Yes	5.48	2.35–12.79	6.78	3.21–14.34
Husband’s depression in current pregnancy				
No	1		1	
Yes	2.68	1.16–6.17	3.95	1.79–8.70

**Table 3:** Logistic regression showing factors associated with expectant fathers’ depressive symptoms

Associated factors	Full model		Final model	
	Adjusted OR	CI_95%_	Adjusted OR	CI_95%_
Socioeconomic status				
Living condition				
Own house	1		1	
Rented house	2.67	1.11–6.45	2.53	1.08–5.92
Living with parents/others	0.70	0.20–2.48	0.77	0.23–2.59
Social support				
Family support	0.82	0.72–0.94	0.83	0.76–0.90
Friends support	1.03	0.91–1.16	–	
Significant other support	1.04	0.95–1.13	–	
Partner relationship				
Relationship with wife				
Good	1			
Poor	1.91	0.72–5.06	–	
Help wife in household chores				
No	1			
Yes	0.51	0.17–1.57	–	
Pregnancy related indicator				
Son preference				
No	1		1	
Yes	2.35	1.13–4.86	2.32	1.15–4.67
Stressful life event				
No	1		1	
Yes	2.62	1.25–5.47	2.92	1.45–5.85
History of mental illness				
Depression in wife’s earlier pregnancy				
No	1		1	
Yes	21.26	6.34–71.32	19.69	6.66–58.23
Wife’s depression in current pregnancy				
No	1			
Yes	2.40	0.96–5.97	–	

The adjusted logistic regression models also identifed the determinants for AAS. The risk of AAS increased for expectant mothers with living condition (living with parents/in-laws), high family support, poor relationship with husband, restriction during preganacy, and stressful life event (Table 4). The risk of AAS for expectant fathers decreased if he supported his wife in household chores, and increased with stressful life event (Table 5). There was no association between AAS and the variation of study sites. No interaction effect of explanatory variables was observed.

**Table 4:** Logistic regression model showing determinants of expectant mothers’ anxiety symptoms

Associated factors	Full model		Final model	
	Adjusted OR	CI_95%_	Adjusted OR	CI_95%_
Socioeconomic status				
Site				
East coast	1			
West coast	1.22	0.87–1.71	–	
Education				
Primary	1			
Secondary	2.42	0.53–11.08	–	
Tertiary	3.53	0.76–16.37	–	
Income earner				
No	1		1	
Yes	0.60	0.42–0.84	0.73	0.53–1.00
Living condition				
Own house	1		1	
Rented house	1.29	0.87–1.91	1.47	1.01–2.13
Living with parents/in-laws	1.82	1.15–2.88	2.18	1.41–3.37
Social support				
Family support	1.05	1.01–1.13	1.07	1.03–1.13
Intimate partner violence				
Physical violence during pregnancy				
No	1			
Yes	3.87	0.94–15.90	–	
Relationship with husband				
Good	1		1	
Poor	1.49	0.98–2.27	1.74	1.17–2.57
Pregnancy related indicators				
Parity				
Multi	1			
Primi	1.08	0.66–1.70	–	
Number of children	0.88	0.74–1.06	–	
Husband’s sex preference				
No	1			
Yes	1.09	0.78–1.53	–	
Restriction during pregnancy				
No	1		1	
Yes	2.22	1.59–3.11	2.30	1.63–3.13
Stressful life event				
No	1		1	
Yes	1.77	1.16–2.68	1.84	1.23–2.75

**Table 5:** Logistic regression examining determinants of expectant fathers’ anxiety symptoms

Associated factors	Full model		Final model	
	Adjusted OR	CI_95%_	Adjusted OR	CI_95%_
Demographic indicator				
Site				
East coast	1			
West coast	1.38	0.79–2.43	–	
Social support				
Friends support	0.94	0.88–1.00	–	
Spousal relations				
Help wife in household chores				
No	1		1	
Yes	0.37	0.17–0.83	0.40	0.18–0.87
Pregnancy related indicator				
Number of children	0.88	0.72–1.07	–	
Stressful life event				
No	1		1	
Yes	2.62	1.56–4.39	2.82	1.70–4.70

## Discussion

The current study is the first study that we are aware of to examine the depressive, anxiety and co-occurring significant symptoms both in expectant mothers and fathers in Malaysia. The result shows that ADS is prevalent in almost one in every eight expectant mothers and one in every 12 expectant fathers in the east and west coasts of Malaysia. Our prevalence of ADS in expectant mothers (12.2%) is in line with the previous report from a systematic review (12%) [6] and from Sabah (13.8%) in Borneo island in Malaysia [26]. However, the prevalence reported in the study is lower than the prevalence from Penang (20%) [38], but higher than the report from Ipoh (8.6%) in the northern part of Malaysia [39]. The heterogeneity observed in the prevalence rates may be potentially related to different assessment methods, multiple cut-off scores, different assessment times, or differences between populations [40].

The prevalence of ADS in expectant fathers (8.4%) in the current study is lower compared to expectant mothers’ rate and identical with the meta-estimate of 8.4% reported in an updated meta-analysis of paternal depression in pregnancy [4]. A prevalence of ADS of 8% in expectant fathers seems rather high, nonetheless Cameron et al. [4] suggested that the prevalence of expectant father’s depressive symptoms during their partners’ pregnancy are nearly twice as high as the rate of depressive symptoms in the general male population. Thus, it indicates that transition to parenthood in men are at high risk of developing depression like women. Moreover, 3.1% of expectant mothers and 1.2% of expectant fathers had an episode of severe depression. These expectant mothers and fathers should be closely monitored and supported after birth, since not only the presence of ADS increases the risk of depressive symptoms after birth, also the severity of ADS matters [41].

This study reported that AAS occurred in just over 1 in 4 expectant mothers and 1 in 10 expectant fathers indicating that AAS is more common than ADS both in expectant mothers and fathers during the third trimester of pregnancy as expected from literature review [1]. A recent meta-analysis conducted in 34 countries (including Malaysia) reported a mean weighted prevalence of AAS of 24.6% in expectant mothers during the third trimester, and this prevalence is higher in LMIC than in high-income countries [42]. The current study confirms the meta-analysis results as the prevalence of AAS was high in Malaysia, and in line with the prevalence in Bangladeshi expectant mothers (29.4%) during their third trimester of pregnancy [23].

The prevalence of co-occurring significant symptoms in expectant mothers was double (8%) than that for expectant fathers (4%). Consistent with Austin et al. [43], we found that two-thirds of the expectant mothers with ADS also experienced anxiety symptoms. Our prevalence is compatible with a meta-estimate of comorbid symptoms of 9.5% in expectant mothers across all trimesters from 30 different countries [44], and 10% in expectant mothers and 3% in expectant fathers during third trimester of pregnancy from Portugal [1]. However, we found a higher rate compared with 4% in expectant mothers from Vietnam [45].

Systematic reviews assessed stressful life event and lack of social support as important risk factors for ADS and AAS [2, 8, 25]. The positive association of stressful life events was pronounced in our study on the outcome of ADS in expectant mothers and fathers, and AAS in expectant mothers not expectant fathers. Pregnancy is a well-recognized time of stress because of potential changes and challenges, and the occurrence of one or more stressful events can lead to an increase in the probability that mothers and fathers experience psychiatric morbidities [25]. However, the study highlighted the importance of perceived social/family support in protecting expectant mothers and fathers from ADS and adds to the prior studies from Malaysia during ante [38] and postpartum period [29]. Several studies indicate social/family support during pregnancy and childbirth may be of special significance in the context of traditional Asian society, including in Malaysia [29, 46]. Adequate support may act as a buffer against the harmful effect of other stressors and difficulties experienced in the transition to parenthood, protecting parental mental health [25]. Surprisingly, this study shows that expectant mothers living in extended families together with parents or in-laws with high family support were more likely to experience AAS, compared to expectant mothers cohabiting with their partners in their own houses. Similar association with depressive symptoms was reported from Italy [47] and Malaysia [29]. Azidah et al. [29] clarified that mothers who had good social support involved in traditional practices related to birth, were more likely to show depressive symptoms compared to mothers who did not follow traditional practices [29]. These findings contradicted the view that traditional practices with family support related to birth is protective against depressive symptoms [46]. Rashid and Mohd [38] explained that in a contemporary society mothers might feel pressure to carry out traditional activities in which they no longer believed in.

Despite a low prevalence of IPV ever (2.6%) and physical abuse during the current pregnancy (1.3%), IPV was found to be one of the strongest predictors of ADS in the current study. There are robust evidences of the adverse impact of IPV on women’s mental health [9], and on depression in particular during the perinatal period [48], when a mother is more dependent. IPV during pregnancy has serious consequences for both the mother and her unborn baby, such as miscarriage, risk of low birth-weight, pre-term labor, and fetal death [48]. Consistent with other research [2, 49], this study shows that poor relationship with husband is another strong predictor of ADS in expectant mothers. Although 19% of the study expectant mothers acknowledged poor relationship with their husbands, its impact on women’s mental health is clearly noticeable in their increased likelihood of reporting depressive symptoms. Risk is also increased among the expectant fathers with the expected sex of the unborn child. The gender preference in favour of a boy has been deeply ingrained in some Asian societies [12], possibly more in expectant fathers than in expectant mothers. Although the participants were not aware about the sex of the unborn child, surprisingly more than one-quarter of expectant fathers in both sites preferred for a boy child. Gender preference and the manifestations of sex preferential behavior are expected to be intrinsically influenced by family, community, and sociocultural norms and expectations, and should not be considered solely the result of parental desires [12].

Previous depression at any time during lifetime and depression during previous pregnancies have been recognised as potential predictors for ADS [2, 8, 25]. In our study, we found only depression in earlier pregnancy both among expectant mothers and fathers, husband’s depression in current pregnancy in expectant mothers, but not previous depression in life predicted ADS at third trimester of pregnancy. The association between ADS in expectant mothers and having a depressed husband has been confirmed by Nath et al. [40], which indicates that the family may be more vulnerable if men, who often is the breadwinner in the family becomes ill. There are growing evidences that depression either in expectant mothers or in expectant fathers, increases the likelihood for both to develop ADS [2, 5].

Studies that have examined the association between socioeconomic status and depressive and anxiety symptoms have reported contradictory results [2, 5, 8, 9]. Contrasting findings reported by Wee et al. [2] and Paulson and Bazemore [5] that no association between ADS/AAS and socioeconomic factors was observed either in expectant mothers or in expectant fathers in the current study. However, consistent with a systematic review by Biaggi et al. [25], our study indicates that expectant mothers are protected against AAS if they are income earner, and expectant fathers are more likely to experience ADS if they live in rented houses. Nath et al. [40] explained that the economy may not be directly associated with ADS/AAS, but it may moderate the relationship between other risk factors and ADS/AAS in expectant mothers and fathers. Contrary to prior research [50], this study did not find any association between ADS/AAS and deviances associated with urbanization. This may be because the sampling was not implemented with independent sample to compare between two study sites.

Several methodological limitations should be considered when interpreting the findings. EPDS is known to have a high rate of false positive as the detection of depression can be impeded by respondent’s culture, gender, and/or predominance of somatic symptoms. However, the positive predictive value at the recommended cut-off point was 80% in the validation study in Malaysia [34], indicating that 80% of the cases might be correctly diagnosed. The DASS anxiety scale has been administered in isolation, which may affect the psychometrics of the scale. Instruments used to measure IPV and stressful life event were not validated. However, translation and back translation of the items of those instruments were done by two bilingual social science researchers. Previous depressive symptoms were identified subjectively by asking question if the respondents were encountered similar depressive symptoms ever in lifetime and in earlier pregnancies, may introduce bias through over-estimation. In the case of reporting physical violence, expectant mothers may not have reported the actual scenario due to its sensitive nature. Several other important variables were not controlled for, such as physical illness, gynaecological morbidities, previous infertility, complications during pregnancy, other mental disorders and smoking. Because of self-reported nature of the interviews, the study recruited only the literate participants that may create a selection bias against those with low education or who have literacy difficulties. Moreover, for highly urban areas, such as Selangor state, there is a greater concern about nonresponse bias, since response rates are lower among expectant fathers; they are not only harder to contact but also less likely to cooperate even after contact. As the study was carried out in purposively selected health clinics in two states of east and west coasts of Malaysia, the findings cannot be generalised.

## Conclusion

Our study confirms that ADS, AAS and co-occurring significant symptoms during the third trimester of pregnancy are common in both expectant mothers and fathers, and that stressful life events, IPV, poor relationship with husband, lack of perceived social/family support, depression in earlier pregnancy, and sex preference for unborn child (expectant fathers only) are independent factors associated with ADS and AAS. The main implication of the results is to integrate a couple-focused screening intervention into the antenatal care services to prevent ADS, AAS and co-occurring significant symptoms. Because of the high costs associated with clinical interviews, research suggested to use screening instruments to identify psychiatric morbidities in LMIC. All pregnant women and their accompanying husbands attending the health clinics for antenatal check-up should be screened for ADS and AAS by the clinic nurses using the locally validated EPDS and DASS-Anxiety. The more structured clinical interview can be followed only for those who screen positive, both expectant mothers and fathers. Moreover, the policies aimed at referring couples with depressive and anxiety symptoms to the nearest health care facilities where psychological treatment is available may help couples in receiving appropriate support.

## Abbreviations

AAS: Antepartum anxiety symptoms
ADS: Antepartum depressive symptoms
DASS: Depression, anxiety and stress scale
EPDS: Edinburgh postnatal depressive scale
IPV: Intimate partner violence
LMIC: Low- and middle-income countries
MSPSS: Multidimensional scale of perceived social support
